# Building Social-Ecological System Resilience to Tackle Antimicrobial Resistance Across the One Health Spectrum: Protocol for a Mixed Methods Study

**DOI:** 10.2196/24378

**Published:** 2021-06-10

**Authors:** Irene Anna Lambraki, Shannon Elizabeth Majowicz, Elizabeth Jane Parmley, Didier Wernli, Anaïs Léger, Tiscar Graells, Melanie Cousins, Stephan Harbarth, Carolee Carson, Patrik Henriksson, Max Troell, Peter Søgaard Jørgensen

**Affiliations:** 1 School of Public Health and Health Systems University of Waterloo Waterloo, ON Canada; 2 Department of Population Medicine Ontario Veterinary College University of Guelph Guelph, ON Canada; 3 Global Studies Institute University of Geneva Geneva Switzerland; 4 Global Economic Dynamics and the Biosphere Royal Swedish Academy of Sciences Stockholm Sweden; 5 Stockholm Resilience Centre Stockholm University Stockholm Sweden; 6 Infection Control Programme and WHO Collaborating Centre on Patient Safety Geneva University Hospitals and Faculty of Medicine Geneva Switzerland; 7 Canadian Integrated Program for Antimicrobial Resistance Surveillance Public Health Agency of Canada Guelph, ON Canada; 8 Beijer Institute of Ecological Economics Royal Swedish Academy of Sciences Stockholm Sweden; 9 WorldFish Penang Malaysia

**Keywords:** antimicrobial resistance, One Health, resilience, transdisciplinary, participatory, interventions, systems dynamics, social-ecological system

## Abstract

**Background:**

Antimicrobial resistance (AMR) is an escalating global crisis with serious health, social, and economic consequences. Building social-ecological system resilience to reduce AMR and mitigate its impacts is critical.

**Objective:**

The aim of this study is to compare and assess interventions that address AMR across the One Health spectrum and determine what actions will help to build social and ecological capacity and readiness to sustainably tackle AMR.

**Methods:**

We will apply social-ecological resilience theory to AMR in an explicit One Health context using mixed methods and identify interventions that address AMR and its key pressure antimicrobial use (AMU) identified in the scientific literature and in the gray literature using a web-based survey. Intervention impacts and the factors that challenge or contribute to the success of interventions will be determined, triangulated against expert opinions in participatory workshops and complemented using quantitative time series analyses. We will then identify indicators using regression modeling, which can predict national and regional AMU or AMR dynamics across animal and human health. Together, these analyses will help to quantify the causal loop diagrams (CLDs) of AMR in the European and Southeast Asian food system contexts that are developed by diverse stakeholders in participatory workshops. Then, using these CLDs, the long-term impacts of selected interventions on AMR will be explored under alternate future scenarios via simulation modeling and participatory workshops. A publicly available learning platform housing information about interventions on AMR from a One Health perspective will be developed to help decision makers identify promising interventions for application in their jurisdictions.

**Results:**

To date, 669 interventions have been identified in the scientific literature, 891 participants received a survey invitation, and 4 expert feedback and 4 model-building workshops have been conducted. Time series analysis, regression modeling of national and regional indicators of AMR dynamics, and scenario modeling activities are anticipated to be completed by spring 2022. Ethical approval has been obtained from the University of Waterloo’s Office of Research Ethics (ethics numbers 40519 and 41781).

**Conclusions:**

This paper provides an example of how to study complex problems such as AMR, which require the integration of knowledge across sectors and disciplines to find sustainable solutions. We anticipate that our study will contribute to a better understanding of what actions to take and in what contexts to ensure long-term success in mitigating AMR and its impact and provide useful tools (eg, CLDs, simulation models, and public databases of compiled interventions) to guide management and policy decisions.

**International Registered Report Identifier (IRRID):**

DERR1-10.2196/24378

## Introduction

### Overview

Antimicrobial resistance (AMR) is a global health crisis that impacts the health and well-being of people and is projected to cause significant social and global economic losses [1-4].

AMR weakens the effectiveness of many antimicrobial agents (eg, antibiotics) used to treat infectious diseases in both humans and animals and is hard to contain because resistance spread between humans, animals, and the environment [5,6]. Through a Tripartite Collaboration, the World Health Organization (WHO), the Food and Agriculture Organization (FAO) of the United Nations (UN), and the World Organisation for Animal Health (OIE) have called for a globally coordinated multifaceted strategy, and a *One Health* approach, to sustainably address AMR and protect future generations from a post–antibiotic era [1,7,8].

### A One Health Approach to AMR

One Health, a paradigm and an approach, recognizes how the health of people is connected to the health of the environment and animals. It emphasizes multisector and transdisciplinary collaborations to comprehensively understand issues and develop sustainable solutions to achieve the health and well-being of people, animals, and the environment [4,9-11].

### AMR as a Complex Adaptive System

In addition to needing a One Health approach, AMR also benefits from being viewed through a complex adaptive system (CAS) lens [12]. CASs are open systems that continuously evolve and reorganize in response to environmental disturbances. These systems comprise multiple agents that act independently but are interconnected such that the actions initiated by one agent change the behavior of others in dynamic and often unpredictable ways [12,13]. AMR can be viewed as both the product and a component of an underlying CAS made up of social, ecological, economic, and other factors [14]. For example, bacteria develop resistance naturally through evolutionary processes and rapidly grow in population size, requiring people to live with resistance. Human behavior is the main driver of AMR, particularly through misuse and overuse of antimicrobials. This unnecessary antimicrobial use (AMU), together with increasing global connectivity of people and animals, growing and intensified human, terrestrial livestock and aquaculture populations, and weakened health because of social inequality, accelerates and exacerbates AMR and the emergence of superbugs [15-20]. Understanding how to build capacity within the underlying CAS to manage the complexities inherent in AMR is essential for finding effective solutions.

### Building Social-Ecological System Resilience to Combat AMR

By viewing the factors that impact AMR as a CAS, we can then apply the lens of social-ecological system (SES) resilience, where resilience is the capacity of a system to cope, adapt, or transform itself into a new state, to manage disturbances in its environment [21,22]. Resilience occurs in 3 ways. Preventive resilience reflects the characteristics of a system that operates in a sustained, desired state (eg, a system in which low AMU or AMR exists). Control resilience is an attribute of a system that can revert to a desired state after a disturbance (eg, a methicillin-resistant *Staphylococcus aureus* hospital outbreak is resolved). Transformability is the capacity of a system to fundamentally change in the face of unsustainable conditions (eg, transforming from a diet high in animal protein to a primarily vegetarian diet to lower AMU and thus AMR). These concepts can be related to ideas of proactive and reactive resilience in supply chain studies [23] and adaptive and coping strategies in ecosystem management [24]. In the context of AMR, a resilient society is one that is able to cope, adapt, and transform in ways that can ensure effective treatment of infections while maintaining or improving economic, social, and environmental health and well-being.

### Understanding How to Build System Resilience Through Interventions

Measuring system resilience to AMR is difficult, although some studies have identified factors associated with the magnitude of the problem [25]. In addition, we can explore and better characterize interventions, including (1) their *effectiveness* in preventing or controlling AMU or AMR, or transforming a system to ensure desired AMU or AMR levels, and (2) the factors that contribute to their success as a way to understand system resilience. As resilience is an emergent property of a system, interventions can be used to measure or test a system’s resilience to AMR. If an intervention is successful, it means that the intervention within the system improved some parameters related to mitigating AMR; in this case, understanding the details of the intervention can help uncover what parts of the underlying system contributed to that resilience. In contrast, if an intervention is not successful, it means that the intervention within the system did not improve parameters related to mitigating AMR; in this case, understanding the details of the intervention can help uncover gaps or deficiencies in the system, including aspects we need to build into the system to bolster resilience (Multimedia Appendix 1 [26-36]).

Understanding what interventions work in what contexts and the factors and conditions that underlie their success will help assess and ultimately improve resilience to AMR. However, limited knowledge exists in this area, particularly an understanding of how to build resilience in different contexts (eg, high-income countries vs low- or middle-income countries) [10].

### Aims and Objectives

This paper describes a study that aims to examine the effects of interventions on AMU and AMR and identify the key factors that influence our ability to address AMR. Interventions will target regional, national, and subnational levels (ie, beyond a single setting such as a single hospital or farm), across the One Health spectrum in high-income (ie, Europe) and low- or middle-income (ie, Southeast Asia) regions of the world. We selected these regions because Europe has undertaken several efforts to address AMR [37], Asian countries, including Southeast Asia, are projected to become the largest users of antimicrobials [16,38,39], and these differences will enable a rich exploration of what interventions work, where, and under what conditions.

To address our study aims, we will complete the following objectives:

1. Identify interventions addressing AMU or AMR and determine the factors that challenge or contribute to the success of interventions.
2. Quantify and validate the ability of interventions to prevent or control rising AMU or AMR or transform the system from persistently high to lower levels of AMU or AMR.
3. Assess whether the types of national and regional indicators that are currently available can predict national AMU and AMR trends across animal and human health.
4. Create causal loop diagrams (CLDs) that depict the system of factors that influence AMR in a high-income region (ie, Europe) and a low- or middle-income region (Southeast Asia).
5. Describe the potential long-term impacts of select interventions that aim to reduce AMR under alternate future scenarios.

### Theoretical Framework and Tools

Our study frames AMU and AMR as part of CAS. Within this framework, we will apply 3 different tools from the respective fields of our interdisciplinary research consortium (systems ecology and evolutionary biology, policy and governance, and epidemiology and public health), as follows.

#### SES Resilience Theory

Although ecological resilience stresses the capacity of a system to withstand shock and maintain function, SES resilience theory posits that a system can have varying capacities to cope, adapt, and transform when disturbances or shocks arise in its environment (eg, anthropogenic changes impacting AMR) [21,22,40]. This can positively or negatively impact the provision of services offered by the system (eg, safe food supply and water) to ensure human and ecological health and well-being. This theory has been predominantly used to understand how to enhance system capacity to withstand disturbances in a variety of fields relevant to the environment, and 7 principles have been theorized to influence system resilience [41].

The first 3 principles represent the key SES properties to be managed to enhance resilience: (1) diversity and redundancy, (2) connectivity, and (3) slow variables and feedback. The remaining 4 principles reflect the attributes of the governance system that manages the aforementioned SES properties: (4) understanding CAS (as described in the *Introduction* section); (5) learning and experimentation; (6) broadening participation; and (7) promoting polycentric governance [41] (Multimedia Appendix 2 [41-49]). Some principles of resilience have been previously described and theoretically applied to AMR [43,44,49]. Our study will allow us to determine whether and how these principles apply in the context of AMR and potentially identify additional factors to refine the SES resilience framework.

#### The Driver-Pressure-State-Impact-Response Framework

The Driver-Pressure-State-Impact-Response (DPSIR) framework is an analytical tool for analyzing environmental problems [50]. Adopted by the European Environment Agency, it is widely used to assess and manage environmental problems such as challenges to coastal regions and freshwater bodies [51], and it facilitates the selection of indicators to assess the implementation of different governance responses, such as environmental policies [52].

Within DPSIR, drivers are social activities that can increase or lessen a particular behavior (eg, why humans use antimicrobials to meet public demand for food animals). *Drivers* exert *pressures* (eg, through AMU) that change the *state* of the environment, which corresponds to the level of AMR measured in different pathogens, which leads to an *impact* (eg, an increase in morbidity and mortality among patients and farmed animals or an increase in economic cost), and results in a *response* by society to minimize the impacts by addressing any part of the DPSIR causal chain [53]. Building on this work, we will categorize information about the social (eg, forces that determine the use of antibiotics) and ecological (eg, microbes in aquaculture) factors related to AMR by the DPSIR components, which will allow us to identify the important cause-effect relationships and potential indicators of changing AMU, AMR, and the impact of responses across the One Health spectrum.

#### Causal Loop Diagrams

CLDs are models that help visualize how different factors in a system are related [54]. Systems dynamics, public health and health care, and epidemiology use CLDs to illustrate relationships between explanatory factors and outcomes of interest [55-58]. We will use CLDs to create visual models of the underlying causal structure (factors, connections, and feedbacks) that generates AMR within One Health systems in a high-income region (ie, Europe) and a low-middle-income region (ie, Southeast Asia). These diagrams are useful because they provide a broad context in which decision makers can explore how particular responses and actions may influence the system [59].

## Methods

### Study Approach

**Figure 1:**
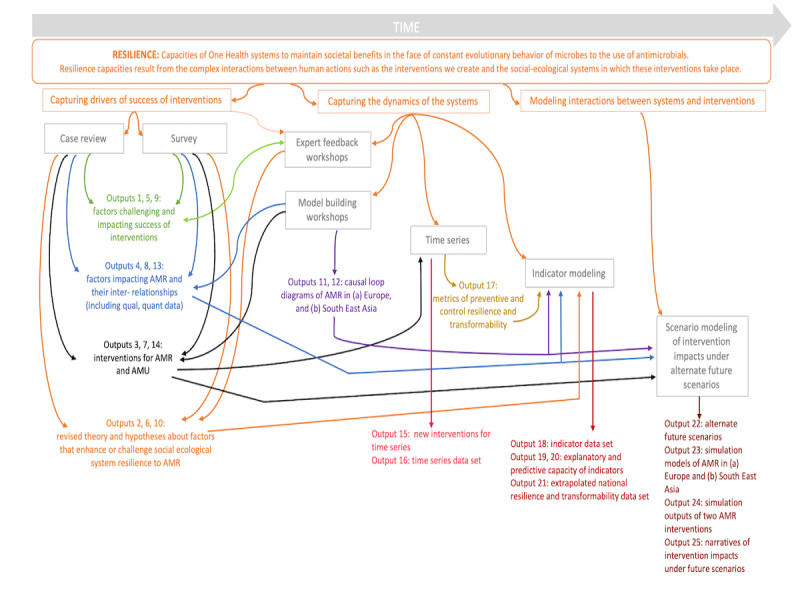
Interconnections between social-ecological system resilience theory, methods, and outputs. AMR: antimicrobial resistance; AMU: antimicrobial use.

We will conduct this study by applying the SES resilience theory for the first time in an explicit One Health and participatory context using mixed methods. Our approach includes 6 interrelated data collection and analysis activities: a review of interventions published in the scientific literature (herein termed *case review*), a web-based survey to collect information about interventions in the gray literature, participatory workshops, time series analysis, regression modeling of indicators, and scenario modeling of interventions. We will identify published and unpublished interventions addressing AMR or its key pressure AMU via a case review and web-based survey. Intervention impacts and the factors that challenge or contribute to the success of interventions will be determined, findings triangulated against expert opinion during participatory workshops, and complemented using time series analysis methods. Regression modeling to identify indicators that can predict national AMU and AMR dynamics across animal and human health will follow. We will then bring AMR experts (eg, physicians and veterinarians) and experts in other content areas (eg, economics and trade and consumer advocacy) who are not traditionally engaged in discussions about AMR together in participatory workshops to create CLDs of the factors influencing AMR in the European and Southeast Asian food systems. Using relevant data collected throughout our study, we will quantify and convert these CLDs into compartment models to simulate the long-term impacts of selected interventions on AMR under alternate future scenarios, re-engage the aforementioned workshop participants to validate simulation findings, and explore the actions and conditions that decision makers should consider to sustainably limit AMR under each future scenario. Figure 1 shows a visual illustration of our approach, including how theory, methods, and associated outputs interlink. We plan to consolidate our work into an evolving web-based learning platform that will house interventions related to AMR from a One Health perspective and be publicly available for decision makers to choose the interventions most likely to build long-term resilience to the challenge of AMR [60].

### Case Review

#### Identifying and Screening Interventions

We will search for interventions addressing AMU or AMR published at any time in the scientific literature using indexed search terms in PubMed and Scopus. The titles and abstracts of each publication will be screened for relevance, and for further screening, retained articles will be read in full. We will include articles that focus on interventions addressing AMU or AMR at subnational, national, or regional levels and exclude articles that are theoretical or policy comparisons or focused on recommendations (eg, AMU guidelines). Additional articles will be identified through reference lists of retained publications and articles recommended by the members of our research consortium. To manage the anticipated large volume of relevant interventions, we will focus on interventions that target important One Health organisms that are most likely to cross between human, animal, and environmental systems and can cause disease in humans and animals (ie, *Escherichia coli*).

#### Data Extraction

We will use a data extraction framework that is underpinned by the SES resilience theory to extract information about each intervention, including the (1) social system (actors, sectors, and any institutional settings involved with the intervention), (2) bioecological system (microorganisms, intervention targets, resistance of the microorganisms, host population or substrate, and the ecology of transmission), (3) triggers and goals of the intervention (what catalyzed the intervention, intervention aims, and strategies used), (4) implementation and governance of the intervention (the types of sectors or institutions responsible for the intervention and the techniques used to enhance intervention adoption, implementation, and sustainability), and (5) assessment (intervention outcomes and any reported factors challenging or contributing to the success of the intervention) [61]. Two researchers (AL and TG) will independently search, screen, and extract data from retained interventions and assess the study quality of interventions using the Grading of Recommendations, Assessment, Development, and Evaluation system [62]. A third researcher (DW) will review all extracted data and the assessment of study quality to ensure consistency in how data are coded. Discrepancies will be resolved through a consensus.

#### Analysis

Analysis will involve coding data from each intervention against the 7 principles of the SES resilience theory, highlighted earlier under the *Theoretical Framework and Tools* section, and the additional factors that emerge from the data and are not already captured by this theory, to identify the factors that challenge or contribute to the success of interventions. Where quantitative data exist, appropriate statistics will be applied (eg, nonparametric statistics to analyze trends in the reviewed interventions and conduct meta-analyses).

We will determine the factors that cross-cut interventions and then categorize interventions based on (1) an intervention’s success in achieving intended outcomes with recognition that a publication bias toward *successful interventions* likely exists, (2) high-income versus low- or middle-income contexts (eg, Europe or Southeast Asia), and (3) settings (eg, aquaculture or community) to determine if any factors that challenge or contribute to the success of interventions differ by context. These factors will then be compiled and compared with the SES resilience theory, further characterizing the 7 resilience principles highlighted under the *Theoretical Framework and Tools* section, adding any additional factors that emerge from the data, and ultimately revising the theory about what factors enhance system resilience to AMR.

#### Case Review Outputs

The following lists the outputs for the case study for use in other data collection or analysis study activities: (1) factors that challenge or contribute to the success of interventions that address AMU or AMR found in published scientific literature; (2) revised theory and hypotheses about the factors that enhance or challenge SES resilience to AMR based on case review findings; (3) a list of interventions identified in the published scientific literature that have shown success or less or partial success in preventing or controlling rising AMU or AMR and in transforming a system to low AMU or AMR; and (4) if available, a list of factors that contribute to AMU or AMR and any qualitative or quantitative data that describe identified factors and any relationships between factors.

### Web-Based Survey

A voluntary web-based survey will be used to collect interventions that address AMU or AMR in the gray literature to expand the understanding of factors that lead to the success of interventions beyond case review findings.

#### Sampling and Recruitment

As the survey is an environmental scan of existing interventions with no hypotheses to be tested, no sample size calculation is necessary. All survey activities will adhere to the approved procedures outlined by the University of Waterloo Research Ethics Committee. To identify potential survey participants, we will develop a matrix of regions based on the WHO’s definition of regions (Africa, Americas, Southeast Asia, Europe, Eastern Mediterranean, and Western Pacific) [63] and populate it with individuals from human, animal, and environmental sectors per region who either work on AMR or do not work on AMR but work in industries that may impact it (eg, food-producing industry) as identified through (1) the WHO library of AMR National Action Plans [64]; (2) the research consortium’s networks (eg, OIE or WHO); (3) web-based search engines (eg, Google); and (4) websites of professional, governmental, nongovernmental, and industry organizations. Contact information will be obtained only from public sources (eg, websites). Potential participants listed in the matrix will be invited to participate in the study via email, which will contain a nonanonymous survey link. Up to 3 reminder emails will be sent. We will announce the survey via a promotional message that will introduce the study and include an anonymous survey link through the mailing lists of national (eg, STRAMA [65]) and global (eg, ReACT [66]) AMR networks. The survey link will direct potential participants to the study information letter in the web-based survey. This letter identifies the study purpose, study investigators, the estimated time to complete reporting one intervention, data storage, and data protection measures that involve replacing personal identifiers from survey responses with an identification code and storing this code and personal information separately on a secure password-protected platform. Potential participants will provide informed consent by clicking *yes* or *no* to participate before starting the survey. Data collection will cease when (1) no new interventions are reported across participants or (2) within 1 month of the survey implementation, whichever comes first.

#### Survey Development, Pretesting, and Implementation

This survey will collect the same information as the data extraction framework described under *Case Review*, including the following: (1) social system, (2) bioecological system, (3) triggers and goals of the intervention, (4) implementation and governance of the intervention, and (5) assessment. One question will be presented per screen, and the *not applicable* response option will be offered. On the basis of input from the type of people that will complete the survey, we will ask survey respondents to coordinate reporting with their collaborators to ensure that a given intervention is reported once, share documents relevant to each reported intervention, and provide permission to include their reported intervention(s) and contact information in the web-based learning platform [60].

Up to 5 individuals, identified by the research consortium as highly knowledgeable about AMU and AMR interventions, will be invited through email to pretest the web-based survey and complete a 30-minute follow-up telephone interview to determine if they interpret and answer questions as intended and obtain their impressions about the web-based survey and its contents. Research team members will test the functionality of the survey. Feedback will inform survey revisions.

#### Analysis

The analysis will involve coding reported intervention data against the 7 principles of SES resilience theory and any additional factors that emerge from the data to determine the factors that challenge or contribute to the success of interventions. Where quantitative data exist, appropriate statistics will be applied (eg, nonparametric statistics to analyze trends in the reviewed interventions and conduct meta-analyses). We will determine the factors that cross-cut interventions and then categorize interventions based on (1) an intervention’s success in achieving intended outcomes with recognition that a publication bias toward *successful interventions* likely exists, (2) high-income versus low- or middle-income contexts (eg, Europe or Southeast Asia), and (3) settings (eg, aquaculture or community) to determine factors unique to these contexts that challenge or foster the success of interventions. These factors will be compiled and compared with the SES resilience theory, providing information that further characterizes the 7 principles highlighted under the *Theoretical Framework and Tools* section, adding any additional factors that emerge from the data, and developing hypotheses about what factors enhance system resilience to AMR. We will use the Checklist for Reporting Results of Internet E-Surveys as a guide to inform the reporting of methods and analysis in future publications of the web-based survey [67].

#### Survey Outputs

The following lists the outputs from the web-based survey for use in other data collection and analysis study activities: (5) list of factors that challenge or contribute to the success of interventions in gray literature; (6) revised theory and hypotheses about the factors that enhance or challenge SES resilience to AMR based on survey findings; (7) a list of interventions that have shown success or less or partial success in preventing or controlling rising levels of AMU or AMR and transforming a system to lowered levels of AMU or AMR from the gray literature; and (8) if available, a list of factors that contribute to AMU or AMR and any qualitative or quantitative data that describe identified factors and any relationships between factors.

### Participatory Workshops

#### Overview

Workshops are a type of research methodology that brings groups of people together to learn from one another, problem-solve, or innovate and, through the process, generate integrated knowledge about a domain of interest to fulfill a research purpose [68]. We will bring together diverse perspectives in 2 types of in-person participatory workshops: (1) expert feedback workshops to triangulate the results of the case review (output 1) and a survey (output 5) against expert opinions to determine factors that challenge or contribute to the success of interventions in a high-income region (Europe) and low- or middle-income region (Southeast Asia) and (2) model-building workshops to develop CLDs of the factors influencing AMR in the food system of a high-income region (Europe) and low- or middle-income region (Southeast Asia). Workshops will last for 8 days (4 days in Europe and 4 days in Southeast Asia). As our expert invitees may be relevant for both types of workshops, we plan to carry out the workshops together to maximize attendance.

#### Procedures Common to Both Expert Feedback and Model-Building Workshops

For both workshop types, we will select participants from across Europe and Southeast Asia who represent diverse perspectives. For the expert feedback workshops, we plan to recruit 12 to 28 participants in total (n=6-14 in Europe and n=6-14 in Southeast Asia) representing the human, animal, and environmental sectors. These participants will have a broad understanding of interventions addressing AMU or AMR in animals, humans, or the environment and will include stakeholders who work directly with end users (eg, farmers). For the model-building workshops, we plan to recruit new participants for each workshop session and aim for 24 to 56 different participants in total (n=12-28 in Europe and n=12-28 in Southeast Asia) that ideally represent an equal distribution of experts in AMR (eg, physicians, epidemiologists, or veterinarians) and experts in other areas of content (eg, farmers, food retailers, consumer advocates, pharmacists, trade and economics, food security, or conservationists), who may not usually be considered in discussions about AMR, but may directly or indirectly impact AMR. AMR experts will provide a deeper understanding of the AMR context in Europe’s and Southeast Asia’s food systems whereas nontraditional experts will help to advance the current understanding of a broader range of factors that may generate AMR, beyond what AMR experts already know. Participants will be identified from (1) the research consortium’s networks and (2) web-based search engines (eg, Google), professional networking sites (eg, LinkedIn), social media sites (eg, Twitter or LinkedIn), and websites of professional, governmental, nongovernmental, and industry organizations that address AMR in human, animal, and environmental sectors.

Both workshop types will be led by a facilitator, guided by a semistructured interview guide, audio recorded, and involving note takers to record discussion points. We anticipate that recruited participants will speak English, and we will provide translation if needed.

Key informant interviews may be conducted after both workshop types to capture additional perspectives identified during workshop discussions as important to fill knowledge gaps. Interviews will follow the same semistructured interview guide used in the workshop and be conducted over the phone, 60 minutes in duration, and audio recorded. All participants will receive via email an anonymous and voluntary web-based survey to evaluate the extent to which the workshop or interview approach fosters open dialogue and learning and participants’ intentions to apply what they will learn in their work. Data will be descriptively analyzed, and findings will inform improvements to future workshops (eg, scenario modeling workshops described later).

#### Expert Feedback Workshop Procedures and Analysis

Workshop sessions will begin by welcoming participants and describing the workshop objectives and agenda. After presenting the findings from the case review (output 1) and web-based survey (output 5), experts will discuss whether they agree or disagree with the findings and identify any missing factors that may challenge or contribute to intervention success based on their expert opinion. Discussions will continue until no new information emerges.

Transcripts from workshops and any interviews will be coded and compared across coders (TG, IAL, and MC) for consistency and thematically analyzed to identify the factors that challenge or contribute to the success of interventions. We will compare these findings to outputs 1 and 2 from the case review and outputs 5 and 6 from the survey to further refine the theoretical framework of factors influencing the success of interventions and draw hypotheses about what factors enhance system resilience to AMR.

#### Expert Feedback Workshop Outputs

The following lists the outputs from the expert feedback workshops for use in other data collection and analysis study activities: (9) factors that challenge or contribute to the success of interventions based on expert opinion and (10) revised theory and hypotheses about factors that enhance or challenge SES resilience to AMR.

#### Model-Building Workshop Procedures and Analysis

Workshops will begin by welcoming participants, describing the workshop objectives and agenda, and providing background information on AMR as participants will have varying levels of understanding about the topic. The facilitator will introduce a previously developed CLD of factors influencing AMR in Canada’s food system [14], and participants will brainstorm what factors and relationships to add, remove, or change in the model to reflect AMR in the European or Southeast Asian food system contexts. They will also identify leverage points and actions that may shift these food systems toward more sustainable management of AMR. Changes to the model will continue until the participants have no new information to share. Although no system model is 100% correct because the external landscape is ever changing, the CLDs will reflect the best estimation of the dynamics that influence AMR in Europe and Southeast Asia based on the perspectives of the participants at the time of data collection [55].

To build these CLDs, 1 researcher (MC) will extract every factor from the workshop and any interview transcripts—any descriptions about the direction and nature of relationships between factors (ie, positive and negative associations) and potential interventions to address AMU or AMR mentioned by participants. Missing information will be added to the model produced during the workshops using appropriate software. Each relationship between factors will be depicted by an arrow (→) to denote its direction, and a positive (+) or negative (−) sign will be added to the arrow to illustrate the nature of the relationship. A positive relationship indicates that 2 factors are moving in the same direction (eg, an increase in X leads to an increase in Y, or a decrease in X leads to a decrease in Y). A negative relationship indicates that 2 factors move in opposite directions (an increase in X leads to a decrease in Y, or a decrease in X leads to an increase in Y). Researchers (IAL, SEM, JP, CC, and MC) will review the transcripts and model and discuss areas requiring clarification regarding the placement of factors and relationships. Disagreements will be resolved through consensus. Each factor in each CLD will be measurable (eg, AMU increases or decreases) and written as a short textual phrase. Transcripts will be thematically analyzed (IAL) to describe key findings from the workshops. The CLDs and a workshop summary of key themes will be sent to participants for validation via feedback.

#### Model-Building Workshop Outputs

The following lists the outputs from the model-building workshops for use in other data collection and analysis study activities: (11) CLD of factors influencing AMR in the European food system; (12) CLD of factors influencing AMR in the Southeast Asian food system; (13) qualitative or quantitative data that describe each factor and relationship in the CLDs; and (14) a list of potentially promising interventions to address AMR.

### Time Series

#### Overview

The aim of the time series analysis is to complement findings from case reviews, surveys, and expert feedback workshops by quantifying resilience and transformations using time series analysis methods. We will quantify (1) preventive resilience, approximated by the stability of resistance levels over time; (2) control resilience, approximated by the ability to lower resistance levels following a relatively large increase; and (3) transformability, approximated by the size and duration of reduction in the specific metric.

We will identify different types of interventions (prevention, control, and transformability) with quantitative data to run time series analyses. By creating and applying metrics of resilience and transformability to standardized data formats, a more objective comparison will be developed and can be applied in the future to standard time series. The widespread quantification of resilience in high-frequency time series is challenged by the limited availability of data, different formats, and separating, for example, internal seasonal dynamics from external shocks. We will overcome these challenges by devising metrics designed to standardize reporting formats and annual time series of at least 10 years of length. The methods involve (1) specification of the metrics, (2) data collection and analysis, (3) sensitivity analysis, and (4) cross-validation.

#### Data

We will use human data from national and regional authorities such as ResistanceMap [69] and the European Center for Disease Control [70]. We will analyze metrics annually for the rates of AMU and AMR (data parameters) in humans and animals from high-income regions (eg, Europe) and low- or middle-income regions (eg, Southeast Asia). For the animal side, standardized and reliable data are limited but will test methods such as the Centers for Disease Control’s National AMR Monitoring System for Enteric Bacteria [71]. We will limit our analyses of AMU to drug classes of relevance to *E coli*, an important One Health organism that can live in different places (eg, animals, people, or soil) and can cause diseases in humans and animals.

#### Analysis

##### Metrics

Our method quantifies stylized metrics as proxies for the 3 types of resilience and metrics that can be varied in terms of 2 or more threshold values. For example, the adapted control resilience method quantifies the years to achieve an x% reduction in AMU and AMR after a y% increase over a 1- to 5-year period. By varying y and x over the observed variation, a bivariate density distribution is produced, which can be analyzed in terms of quantiles and compared using standard nonparametric or parametric statistics, depending on skew and sample size. Preventive resilience measures the x-year increase in AMU and AMR and can, in contrast to control resilience and transformative success, be applied as a rolling metric to the time series. Transformations will be quantified in terms of duration and proportional decrease following 3 to 5 years of stable high values. For both resilience and transformation metrics, we will apply the metrics to (1) a general set of time series and (2) a subset of time series where we know of specific interventions from the case review and web-based survey (outputs 3 and 7).

##### Sensitivity Analyses

We will explore the sensitivity of measuring preventive resilience in absolute versus relative terms, the latter by accounting for the mean level of AMR. Similarly, control resilience can be corrected for mean levels of resistance, assuming either a first-order linear or a polynomial relationship between reduction in AMU and reduction in resistance. To overcome the challenge that many countries only have either AMU or AMR data available, but not both, the resilience metrics will be quantified for both AMR and AMU data; the latter serving as a surrogate, for example, by analyzing the ratios of second- to first-line AMU.

##### Validation

We will cross validate the time series analyses against a subset of the particularly well-documented interventions identified through the case review (output 3) and survey (output 7). For interventions at the national or regional level, this can be done through direct comparison with the broader time series analysis. For local-level interventions, we will acquire the relevant local time series data for cross-validation.

#### Time Series Outputs

The following lists the outputs from the time series for use in other data collection or analysis study activities: (15) any new interventions identified for the specific purpose of time series analysis; (16) time series data set; and (17) metrics of preventive and control resilience and transformability.

### Regression Modeling of National and Regional Indicators of AMU, AMR, and Impacts

#### Overview

To assess whether currently available indicators predict national AMR dynamics, we will identify, test, and extrapolate indicators of preventive and control resilience and transformability. This will involve (1) specifying, collecting, and analyzing national and regional indicators of resilience and transformability; (2) quantifying the explanatory and predictive capacity of indicators through regression and simulations; and (3) producing an extrapolated data set of resilience and transformability based on these indicators.

#### Collecting and Specifying Indicators

We will use metrics of preventive and control resilience and transformability from time series analyses (output 17) as a basis for identifying indicators that explain variation in resilience and transformability metrics. National and regional statistics will provide the basis for selecting indicators, and these statistics will be collected from existing databases, such as those hosted by the UN and by contacting regional and national statistics offices. We will select indicators using specific hypotheses about their contribution to resilience and transformability based on findings from the case review (output 2), survey (output 6), expert feedback workshops (output 10), and previous work of coauthors (PSJ and DW) in applying resilience theory and principles in the context of AMR [43,44,49].

We will then code the indicators according to the DPSIR framework.

##### Driver Indicators

Context-specific driver indicators will be added to a previously developed DPSIR framework [53] using the list of factors contributing to AMU and AMR from the case review (output 4), survey (output 8), and participatory workshops (outputs 11 and 12). For instance, key distal driver variables are sanitation, hygiene, vaccine coverage, animal (farm) densities, husbandry type, and social norms regarding common pool resources. Key proximate driver variables are disease prevalence and incidence and access to antibiotics. We will collect these indicators from international statistics compiled by the UN, WHO, FAO, OIE, and European Union and where national agencies are needed. A preliminary assessment during ongoing work on the DPSIR framework indicates that data availability is sufficient to cover the countries in the time series.

##### Pressure and State Indicators

We will use data on the key pressure variable (AMU) and the key state variable (AMR) used in the time series.

##### Response Indicators

Response variables will be scored from a review of national or regional policies led by coauthor DW and grouped into categories of preventive responses (addressing drivers), mitigative (addressing AMU), restorative (addressing AMR), and adaptive responses (addressing impacts). For national indicators, we will in part rely on the WHO-FAO-OIE survey of national actions to limit AMR [72].

#### Analysis

The explanatory capacity of indicators will be tested on a training subset of the time series and their predictive capacity tested on the full subset of the time series. On the basis of this analysis, we will use a broader data set of national (and possibly regional) statistics and run regression modeling or simulation modeling to perform an extrapolative analysis predicting the resilience and transformability of countries and regions without the necessary time series data available.

#### Regression Modeling of Indicator Outputs

The following lists the outputs of the regression modeling of national and regional indicators of AMU, AMR, and impacts: (18) data set of social-ecological indicators; (19) explanatory capacity of indicators; (20) predictive capacity of indicators; and (21) extrapolated data set of national resilience and transformability.

### Scenario Modeling

We will conduct 2 types of scenario modeling: (1) mixed methods simulation modeling using fuzzy logic and (2) group-based scenario modeling using a participatory approach [73,74] to explore the range of possible outcomes of selected interventions on AMR in the food system of Europe and Southeast Asia under future alternate scenarios.

#### Mixed Methods Simulation Modeling Procedures

We will use the CLDs of AMR in the European and Southeast Asian food systems (outputs 11 and 12) and fuzzy set theory [75-77] to build 2-compartment models. We will populate these models using quantitative and qualitative data from the case review (output 4), web-based survey (output 8), modeling workshops (output 13), and from published studies and surveillance data and then convert the data to categorical variables (eg, *high*, *medium*, and *low* categories) to create differential equations [75-77] to simulate the development and movement of AMR in the European and Southeast Asian systems using appropriate software. In the absence of data, we will make reasonable assumptions and document them for transparency.

**Figure 2:**
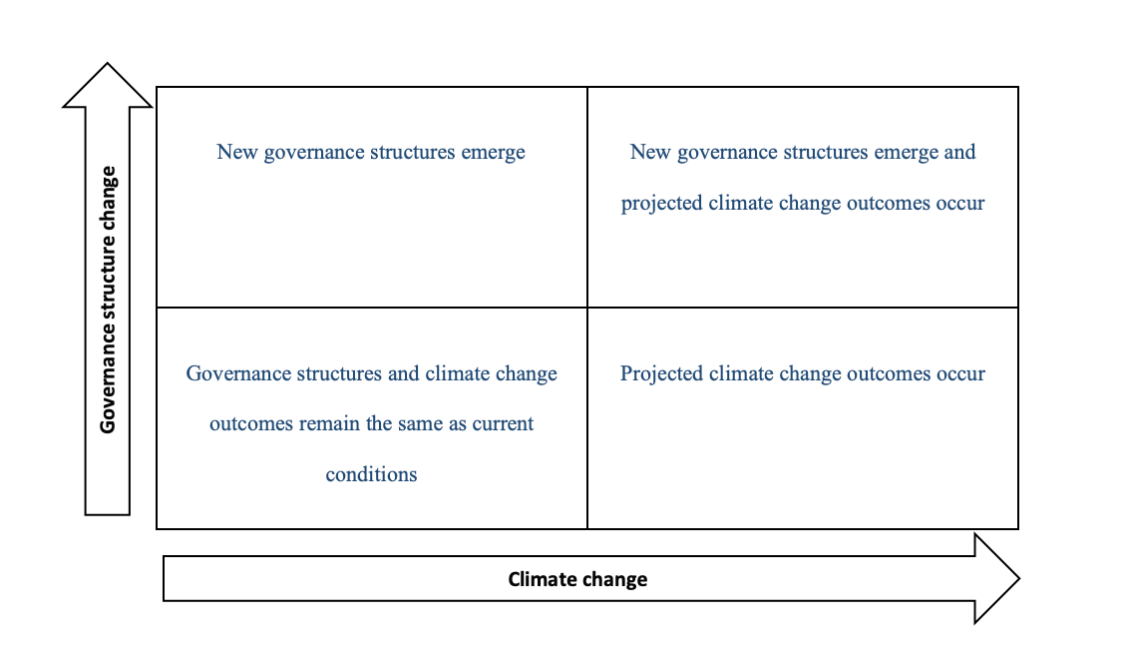
Two-by-two matrix of alternate future scenarios circa 2070 based on governance change and climate change.

We will then use these models to explore the impact of selected interventions on AMR over a 50-year timeframe under alternate future scenarios. To select these interventions, our multidisciplinary research consortium will independently rank the list of interventions from the case review (output 3), survey (output 7), and modeling workshops (output 14) from most to least promising and come to an agreement about the top 2 interventions that may impact AMR. To develop our scenarios, we will construct a two-by-two matrix with climate change on one axis and governance structure change on the other axis, two key factors likely to impact the food system over time, to produce the 4 future scenarios illustrated in Figure 2.

As a limited amount of data at the European or Southeast Asian food system scale make it difficult to validate the simulation model against a data set, we will take the simulation outputs to the group-based scenario workshops for participant validation [78,79]. If there are insufficient data to construct a mixed methods simulation model, we will only conduct group-based modeling workshops.

#### Group-Based Scenario Modeling Workshop Recruitment and Procedures

A total of 4 virtual workshops (2 in Europe and 2 in Southeast Asia) lasting 4 days will be conducted. These workshops will bring together diverse perspectives to (1) validate the model and intervention outcomes from the simulation and (2) brainstorm what factors and conditions (eg, polycentric governance systems or multisector participation) are necessary to strengthen the potential for selected interventions to combat AMR over 10-, 30-, and 50-year timeframes under the alternate future scenarios in Figure 2 [80]. These timeframes are common with foresight methods (eg, [81,82]).

To increase stakeholder commitment and their potential to apply what they learn about AMR into action, we will invite via email selected individuals who participated in our model-building workshops described earlier for a total of 12 to 30 participants (n=6-15 in Europe and n=6-15 in Southeast Asia). These participants will represent an equal distribution of experts in AMR (eg, epidemiologists, veterinarians, or physicians) and other areas that may directly or indirectly impact AMR (eg, corporate food industry, trade and economics, or consumer advocacy). If a participant is unavailable, we will identify and approach a new individual representing a similar perspective using the sampling procedures described for the model-building participatory workshops.

Workshops will be led by a facilitator, guided by a semistructured interview guide, and audio recorded. Members of our research consortium will take notes to record the discussion points. After the welcome, introduction, and overview sessions of workshop objectives, the facilitator will present the simulation model and how the 2 interventions impact AMR over a 50-year horizon under various scenarios. Participants will then discuss whether the model and intervention outcomes align with their expert opinions about how the system should behave to validate the model. Through facilitated discussions, participants will also discuss (1) why they believe each intervention will impact AMR under future scenarios, (2) what barriers and challenges may impact intervention success, and (3) what actions and supports (eg, resources, communication systems, or actors) are necessary to ensure intervention success and circumvent barriers and negative consequences over time. Participants will be encouraged to bring forth all ideas until no new information emerges.

#### Analysis

A researcher (MC) will extract data from transcribed workshop audio recordings to determine whether participants agree with findings from the simulation modeling and any adjustments made to the model about each intervention’s probable impact(s) under each scenario of the future in the European and Southeast Asian context. A researcher (IAL) will conduct a thematic analysis and develop narratives that describe how each intervention will behave under each alternate future scenario, and the factors and conditions that should be implemented to sustainably mitigate AMR while maintaining social, economic, and environmental health over time, based on participant feedback.

#### Scenario Modeling Outputs

The following lists the outputs from the scenario modeling activities: (22) scenarios of alternate futures; (23) simulation model of AMR in the European and Southeast Asian food system; (24) simulation outputs of the impacts of 2 interventions on AMR; and (25) narratives that describe intervention impacts under alternate future scenarios.

## Results

### Ethics Approval

Ethics approval for activities involving participants has been granted by the University of Waterloo’s Office of Research Ethics (ethics clearance numbers 40519 and 41781).

### Case Review

A total of 669 interventions have been identified. In addition, 42 interventions specifically targeting *E coli* were analyzed in full based on the SES resilience theory (reporting is underway).

### Web-Based Survey

The survey has been sent to 891 individuals who work on AMR or carry out work that may impact AMR from 6 regions of the world (Africa, n=27; Americas, n=443; Southeast Asia, n=117; Europe, n=249; Eastern Mediterranean, n=12; Western Pacific, n=43). Survey analysis is pending.

### Participatory Workshops

A total of 4 in-person expert feedback workshops that engaged stakeholders (n=8 from Europe; n=6 from Southeast Asia) representing human (n=5), animal (n=4), human and animal (n=3), and environment (n=2) sectors have been completed.

A total of 4 in-person model-building workshops that engaged 32 stakeholders (n=17 from Europe; n=15 from Southeast Asia) representing advocacy (n=2), nutrition, food security, and food safety (n=5), economics and trade (n=2), human medicine (n=5), pharmaceutical (n=3), agricultural food and animal health (n=10), sustainable food innovations (n=2), environment (n=1), peace and leadership (n=1), and law (n=1) perspectives have been conducted. Analysis is underway.

### Time Series

Time series analysis activities are anticipated to be completed by spring 2022.

### Regression Modeling of National and Regional Indicators of AMR Dynamics

Activities are anticipated to be completed by spring 2022.

### Scenario Modeling

Mixed methods simulation modeling activities and 4 virtual group-based scenario modeling workshops and analysis are anticipated to be completed by spring 2022.

## Discussion

### Anticipated Findings and Contributions

To our knowledge, this is the first study to apply SES resilience theory, systems thinking, and a One Health approach to better understand how to sustainably combat AMR. Our study is in progress and is not yet complete. However, we anticipate that our study will help make sense of the diversity of actions to tackle AMR and add to our limited understanding of which actions work under what conditions. We intend to consolidate our findings into a web-based platform that will allow stakeholders to add interventions and use the tool to determine what actions to take in their respective contexts. We also anticipate that through a series of participatory workshops that engage AMR experts and stakeholders who may not usually be engaged in discussions about AMR, our study will produce useful tools (ie, CLDs of AMR in Europe and Southeast Asia, and alternate future scenarios) to help build stakeholder capacity to recognize AMR as a CAS and plan interventions under uncertain future conditions. The time series and regression of indicators analyses will help to gain a better understanding of the relationships among drivers, pressures, states, and responses regarding AMR. In addition, as we quantify and carry out simulation modeling using data from our study and the literature, our study will help to identify data gaps for future research.

### Conclusions

One Health and systems thinking have gained prominence in public health but can be challenging to conduct because they necessitate collaboration and the integration of knowledge from science and practice across different sectors and disciplines. Our protocol provides other researchers with an example of how to apply these approaches to study a complex public health problem such as AMR with an interdisciplinary research team and involving AMR experts and nontraditional stakeholders. In fact, we developed this paper to help our research consortium bridge our disciplinary-specific knowledge, skills methods, and tools and make our processes transparent so that others can learn from our experiences as we implement this mixed methods study.

## Appendix

Multimedia Appendix 1 Examples of how interventions can inform the building of social-ecological system resilience.

Multimedia Appendix 2 Social-ecological system resilience principles.

## Abbreviations

AMR: antimicrobial resistance
AMU: antimicrobial use
CAS: complex adaptive system
CLD: causal loop diagram
DPSIR: Driver-Pressure-State-Impact-Response
FAO: Food and Agriculture Organization
OIE: World Organisation for Animal Health
PI: principal investigator
SES: social-ecological system
UN: United Nations
WHO: World Health Organization
