# Supplementary material for: Building Social-Ecological System Resilience to Tackle Antimicrobial Resistance Across the One Health Spectrum: Protocol for a Mixed Methods Study
Source: JMIR Res Protoc. 2021 Jun 10;10(6):e24378. doi: 10.2196/24378 (PMC8262547; doi:10.2196/24378)
Supplement: Multimedia Appendix 1 [file resprot_v10i6e24378_app1.pdf]

Multimedia Appendix 1: Examples of how interventions can inform how to build SES resilience

The European Union's (EU's) ban on the use of antibiotics as growth promoters in food-animals is considered a successful intervention that reduced certain types of AMR [26], and exploring the features of this intervention may help uncover what parts of the underlying system contributed to resilience. Although unintended consequences to animal health (e.g., increased diarrhea, weight loss in weaning pigs, increased therapeutic use of important human antibiotics in food-animals) did emerge shortly after the ban [27], these issues were mitigated through better animal health management and housing practices, ultimately improving system resilience and reducing reliance on antimicrobials [28].

Compared to the ban described above interventions that limit the use of specific antibiotics appear less effective in reducing AMU, in part because non-restricted medications may replace restricted medications [26]. For example, ceftiofur (a 3rd generation cephalosporin which is in a class considered critically important for human medicine) [29] was used in the Canadian poultry sector to prevent yolk sac infections in young chicks. This use practice temporally mirrored resistance to this drug class in Salmonella found in chickens on farm, retail chicken meat, and Canadians. The Canadian poultry industry eventually instituted a nation-wide ban on the preventive use of ceftiofur in poultry, and resistance levels decreased in both poultry and humans [30]. Over time other antibiotics (gentamicin and lincomycin-spectinomycin) took ceftiofur's place, increasing resistance to these/related drugs in the poultry sector [31]. Exploring the ceftiofur ban provides insights into what parts of the underlying system contributed to or detracted from the system's resilience. While the ban on the clinically significant ceftiofur is a relative success for public health, the introduction of other antibiotics as replacements to

ceftiofur illustrates how banning a specific antibiotic or antibiotic class may require other concomitant changes (e.g., actors in the system adapting by changing where chicks are sourced or housed, to reduce the need for preventative antibiotics) for the system to be resilient and for AMR to reduce. Biosecurity measures to prevent infections on farms [32], and infection prevention and control measures in healthcare settings (e.g. hand hygiene) and in the community (e.g., sanitation and immunization) [33–36], are some examples of other types of interventions demonstrating success in addressing AMR and may provide important insights into what is needed to build resilience to AMR.
