# Supplementary material for: Building Social-Ecological System Resilience to Tackle Antimicrobial Resistance Across the One Health Spectrum: Protocol for a Mixed Methods Study
Source: JMIR Res Protoc. 2021 Jun 10;10(6):e24378. doi: 10.2196/24378 (PMC8262547; doi:10.2196/24378)
Supplement: Multimedia Appendix 2 [file resprot_v10i6e24378_app2.pdf]

Multimedia Appendix 2: SES Resilience Principles

Overview of Social-Ecological System (SES) Resilience Principles

Key social-ecological system properties to be managed to enhance resilience: diversity and redundancy; connectivity; and slow variables and feedback [41]. Diversity and redundancy of actors, species, and other social and ecological elements contributes to system resilience because a diversity in options reduces vulnerabilities in a system, redundancy allows some components to compensate for others in the face of loss or failure, and both increase a system's options for how to respond to disturbances in the environment [41]. For instance, while food supply diversity has improved globally due to trade dependency, many importing countries risk exposure to disturbances (e.g., crop failures) experienced in the few existing exporting countries [42]. Increasing the number of exporting countries may be one step towards mitigating risk and improving food system resilience. Connectivity, the extent to which resources, actors, and species interact, reflects the ease with which impacts can move through a system. High connectivity can assist with recovering from rising AMR in the environment (e.g., through organizations sharing information), but can also facilitate AMR spread (e.g., through transport and international travel) [43]. Feedback and slow variables determine the emergent behaviour of CAS, and managing both enhances a system's ability to cope with disturbances while continuing to produce desired services [41]. AMR emerges from positive feedback between increasing AMU and increasing AMR, which is reducing the effectiveness of antimicrobials, creating pan-resistance for some infections, and shifting society from being able to treat simple infections to one that is less able to do so [44]. Slow variables are factors that change slowly within a system and shape how quickly AMR might develop and spread [41], such as the combination of levels

of disease prevention and control measures, (e.g., sanitation, hygiene, immunizations), types of regulations and prescribing norms among physicians.

The remaining four principles reflect the attributes of the governance system that manages the social-ecological properties above: iv) understanding CASs (as described in the introduction section); v) learning and experimentation; vi) broadening participation; vii) and promoting polycentric governance [41]. Learning, occurs at the individual level, spreads through social interaction to broader levels (e.g., organizations), and contributes to resilience by enabling actors to anticipate, adapt, and evolve in response to changes in the external environment that impact AMR [41]. Experimentation and monitoring facilitate learning via examining how actions change the environment, but participation of a broad range of actors can foster social capital and enable deeper learning processes that can uncover assumptions, practices and worldviews that perpetuate AMR and lead to transformative solutions that enhance system resilience to AMR [41,45,46]. Polycentric governance reflects different (and potentially inter-linked) governing authorities at local to broader levels that make decisions within their specific geographic area or context [47]. This governance system enables the other resilience enhancing principles highlighted above (e.g., through setting goals, instituting monitoring systems to track each principle, creating opportunities for experimentation and broad participation and integrating diverse knowledge [47,48]. Some principles of resilience have been previously described and theoretically applied to AMR [43,44,49]. Our study will allow us to determine whether and how these principles apply in the context of AMR, and potentially identify additional factors to refine the SES resilience framework.
